# A randomized controlled trial of a nurse-led psychological pain solution-focused intervention for depressed inpatients: study protocol

**DOI:** 10.1186/s12912-023-01252-6

**Published:** 2023-04-10

**Authors:** Shu-Yan Chen, Cheng Bian, Yin Cheng, Wei-Wei Zhao, Shi-Rui Yan, Yan-Hong Zhang

**Affiliations:** 1 School of Nursing, Nanjing Medical University, 101 Longmian Avenue, Jiangning District, Nanjing, Jiangsu Province Nanjing, China; 2 The Affiliated Brain Hospital of Nanjing Medical University, Nanjing, China

**Keywords:** Psychological pain, Depression, Nursing intervention, Solution-focused brief therapy, Hope theory, Protocol

## Abstract

**Background:**

Depressed patients commonly experience psychological pain. Research pointed to positive psychological interventions as an effective means of ameliorating psychological pain, although the exact effect is unclear. Based on the hope theory and solution-focused brief therapy (SFBT), this study combines hope theory with solution-focused brief therapy to develop a nurse-led psychological pain solution-focused (PPSF) intervention in depressed patients.

**Methods:**

This is an assessor-blinded randomized controlled trial following the SPIRIT guidance. A total of 84 depressed patients will be recruited from the inpatient wards of a psychiatric hospital and randomly assigned to the control and experimental groups. Patients in the control group will be treated as usual. In contrast, patients in the experimental group will receive 6 sessions of the PPSF intervention for two weeks on top of the routine care. Primary outcomes are psychological pain, hope, cognitive distortions. Secondary outcomes are depression and suicidal ideation. Data will be collected at 5-time points: baseline, 1 and 2 weeks (post-intervention), 1 month (follow up), and 6 months after baseline. Generalized equation evaluation will be used to assess the effectiveness of the PPSF intervention.

**Discussion:**

From a positive psychology perspective, there remains much room for developing psychological pain interventions in depressed patients. SFBT and hope theory are both based on positive psychology. With hope theory as the general framework and SFBT questions as the practical guide, the PPSF intervention program is designed that nursing staff can implement. If the intervention is effective, it will advance the development of psychological pain interventions for patients with depression.

**Trial registration:**

ChiCTR2100048223

## Introduction

Depressive disorder is a mental disorder marked by low mood and lack of pleasure that significantly impacts the quality of life. More than 350 million people worldwide are currently estimated to suffer from depressive disorders [1]. In China, the situation is also not optimistic. In the China Mental Health Survey (CMHS), Huang et al. [2] reported the lifetime prevalence of depression in China as 6.9%, exceeding 54 million patients. Depression is the leading cause of suicide, with a rate about ten times higher than that of the general population [3]. As the most common complaint of patients with depression, psychological pain is a risk factor for suicide and plays a central role in suicidal development [4]. Shneidman’s psychache model proposed that “no psychological pain, no suicide”. In other words, when a person’s psychological pain has become unbearable, he will choose suicide to end the pain [5].

As a complex construct, the definition of psychological pain remains inconsistent. Researchers have proposed different definitions of psychological pain from other points of view. Shneidman [5] defined psychological pain in his theory of suicide as an intense, acute mental state accompanied by feelings of guilt, pain, fear, panic, anxiety, loneliness, and helplessness. By applying grounded theory to the emotional pain of individuals with traumatic experiences, Bolger [6] proposed that psychological pain is an individual’s self-awareness of his or her role in the experience of emotional pain, which is fundamentally characterized by a sense of loss or incompleteness of self. Also, Mee et al. [7] defined psychological pain as a diffuse, subjective experience and distinguished it from physical pain produced by physical stimuli that are usually localized. Synthesizing the above perspectives, we attempted to define psychological pain in the domain of depression as a subjective, intensive, and mixed emotional experience that centers on a sense of suffering due to negative self-concept and incompetent coping based on personhood traits [8].

Increasing evidence points to psychological pain as a prominent symptom in depressed patients that aids in assessing and predicting suicide [9, 10]. In the systematic review, Verrocchio et al. [11] found significant positive associations between psychological pain and suicide in the population with depressive disorder. Also, psychological pain plays a mediating role in many variables (e.g., the severity of depressive symptoms, despair, loneliness, adverse life events) and suicide [12–14]. In addition, significant associations were still found when longitudinally assessing the relationship between psychological pain and suicide in patients with psychiatric disorders [15]. This suggests that psychological pain is a stronger predictor of suicide and further emphasizes that early intervention for psychological pain can help prevent and manage suicide. In China, among patients with depressive disorders, the prevalence of psychological pain was 95.78%, which was moderate or higher overall and significantly higher than normal [16, 17]. Given the severity of the situation, it is essential to provide early intervention for psychological pain in depressed patients who may be at risk for suicide but present with psychological pain rather than intervening after it is clear that the suicide risk has emerged.

### Background

In searching and reading the literature on psychological pain interventions in depressed patients, we found many studies of interventions directly targeting suicidal ideation or suicidal behavior in depressed patients. Still, interventions for psychological pain are only in the beginning stages, which may be related to the destructive nature of suicide. Once individuals commit suicide, it causes severe damage to the individual, family, and society, which is much greater than psychological pain. This explains why most of the previous interventions targeted suicide. Only three studies reporting psychological pain interventions for depression patients were searched in the literature. Songprakun and McCann [18] explored the effects of cognitive behavioral bibliotherapy (CBB) in outpatients with moderate depression and measured psychological pain using the Kessler Psychological Distress Scale. Zou et al. [19] used psychological pain theory-based cognitive therapy (PPTBCT) in depressive patients and measured psychological pain using the Three-Dimensional Psychological Pain Scale. Yu [20] used simplified cognitive behavioral therapy(SCBT) for depressed patients with psychological pain, where psychological pain was measured using the Distress Thermometer (DT). These interventions use different types of cognitive behavioral therapy (CBT) and have decreased psychological pain in patients with depression. The focus of these interventions is to guide patients to reduce negative affect, such as CBB’s modification of negative thoughts, PPTBCT’s questioning of irrational beliefs and SCBT’s discovering of negative emotions and dealing with negative beliefs. During the intervention, patients have been in a chronic passive state for a long time, so the experience of positive effects is relatively ignored. However, promoting positive affect is as important a priority in treating depression as reducing negative affect. Positive psychological interventions do not focus on helping patients cure their pathological symptoms but on enhancing positive affect and positive cognition. There is increasing evidence that positive psychological interventions can improve positive emotions and reduce negative emotions [21, 22]. WHO [23] recommends that trained non-professional mental health staff can provide brief psychological treatment to individuals with depression. As those in close contact with depressed patients, nursing staffs are ideal for implementing the intervention. The nurse-led positive psychological intervention can enable patients to discover their potential strengths and capabilities through interventions. Shi et al. [24] stated that nurse-led positive psychological interventions could alleviate depression and improve well-being. Therefore, there is a need to design a brief and practicable nurse-led intervention from a positive psychology perspective to reduce psychological pain.

Solution Focused Brief Therapy(SFBT) [25] is a positive psychology-based, patient-centered, short-term therapy that focuses on the patient’s strengths and the future rather than problems and the past. It is more important to note that SFBT has the advantages of promoting positive affect, short cycle time, low cost, time-effectiveness, and easy operation. We chose SFBT for the intervention based on the following considerations: First, grounded in positive psychology, SFBT adheres to the principle that the patient is the expert in the treatment process, emphasizing the patient’s resources and successful experiences to build solutions to problems jointly. The nature of psychological pain in depressed patients is a negative emotional experience. The key to improving psychological pain is the cognitive reappraisal of emotional events, which means giving new meaning and understanding to emotional events [26]. SFBT can better cope with psychological pain by enhancing positive affect rather than focusing on the causes of psychological pain. Second, SFBT is a highly effective and brief therapy that results in positive changes within 3–4 sessions [27]. It dramatically reduces expenses; on the other hand, it is more acceptable to patients because of the reduced number of sessions. Third, SFBT is a strengths-oriented, patient-centered intervention that fits with the values and principles of nursing. Questioning techniques such as miracle questions and exception questions in SFBT are important ways to drive change in participants, and this is where it is easy to practice. Evidence [28] shows that nurses who complete a brief 2–3-day SFBT training and use SFBT in clinical settings make positive changes to patients and themselves. Also, a qualitative study [29] indicated that SFBT is a beneficial and implementable intervention in the nursing field and that it can be easily integrated by nurses into their clinical work. Therefore, it is reasonable to assume that SFBT is an implementable and brief pathway to reduce the psychological pain of patients with depression that can be implemented by nurses.

### Theoretical framework

Although SFBT offers simple and easy-to-use question techniques, there is no specific and detailed structure for SFBT, and it is challenging to ensure consistency in the implementation of SFBT content. Hope, as one of the four factors of psychological capital, plays an important role in positive psychology [30, 31]. In addition, hope-based intervention has shown positive effects on patient health recovery across broad clinical practices [32–35]. Hope theory was developed by the positive psychologist Snyder and his colleagues [36]. In recent years, it has been gradually applied in the management of mental diseases, which can enable patients to acquire positive thinking ability, so that patients can face the disease in a positive way and reduce their psychological pain [37–40].

Based on the following considerations, we apply the hope theory to guide the study design. First, hope theory and SFBT fall under the umbrella of positive psychology, emphasizing the individual’s strengths and capabilities and focusing on positive emotions such as hope. In principle, Hope theory and SFBT are compatible. Second, compared to SFBT, hope theory provides a more detailed view that guides better design programs. In contrast, SFBT delivers well-used question techniques such as exception questions, scale questions, and miracle questions. In conclusion, integrating SFBT and hope theory is more beneficial in designing programs that reduce psychological pain.

**Fig. 1:**
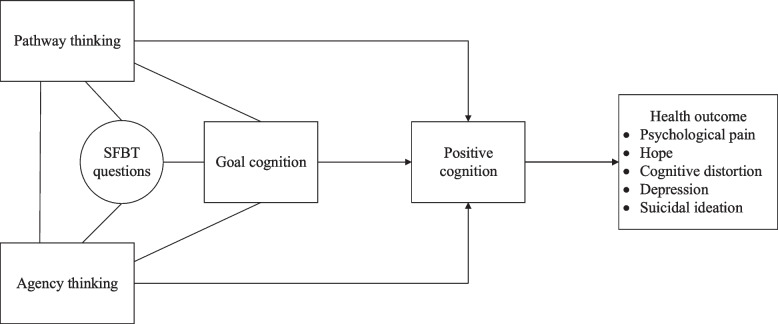
Conceptual framework

Hope theory includes three components: goal cognition, pathway thinking, and agency thinking, arguing that hope refers to cognition derived from a combination of successful path thinking and agency thinking [41]. Hope theory is more conducive to broadening the depth and breadth of psychological pain research as a framework for understanding pain responses [42]. Furthermore, as emphasized by hope theory, hope is an important psychological resource for individuals to cope with pain and buffer individuals against psychopathology [43]. High-hope individuals are equipped with more positive cognition and are better able to develop strategies to cope with pain and difficulties. Hope as a protective factor can be introduced as a positive motivation at different stages of intervention [44]. Based on Hope Theory and SFBT questions, the intervention strategy in our study was developed around the three components of hope theory of goal cognition, pathway thinking, and agency thinking. And SFBT questions were integrated throughout the intervention as a technical tool. This intervention target is acting on positive cognition, thereby helping to reduce psychological pain, cognitive distortion, depression, and suicidal ideation and improve hope. A conceptual framework is developed, as shown in Fig. 1.

Therefore, based on the hope theory to provide a general framework and SFBT questions to provide practical guidance, we developed a new intervention program called the Psychological Pain Solution Focused (PPSF) intervention through a literature review of psychological pain intervention studies. If the program is effective, it could be used by nursing staff for interventions for psychological pain in patients with depressive disorders.

## The study

### Aim

This study aims to develop a study protocol for PPSF to improve psychological pain and other psychological variables in patients with depression and to evaluate its effectiveness.

### Hypotheses

- Primary hypothesis: compared to the control group, patients in the intervention group receiving the PPSF intervention will have significantly lower psychological pain scores, higher hope scores, and cognitive distortion scores.
- Secondary hypothesis: compared to the control group, patients in the intervention group will reduce depression scores and suicidal ideation scores after receiving the PPSF intervention.

### Design

The design of this study will be an assessor-blinded, two-arm, randomized, controlled, equivalence trial with an allocation ratio of 1:1. Since psychological interventions are difficult to blind the participants and the researchers conducting the intervention, this study will be blinded only to the researchers assessing the outcome. The protocol will adhere fully to the SPIRIT 2013 statement.

### Study setting

This study will be carried out in a tertiary-A psychiatric hospital in Nanjing, Jiangsu province, which has 16 wards with 573 psychiatric beds. The hospital admits more than 3000 depressed inpatients per year.

### Participants

#### Inclusion criteria

Patients meeting the following criteria will be included: (1) meet the ICD-10 diagnostic criteria for depression; (2) are aged 18–60 years; (3) have depression scores ≥ 20 measured as measured using the 24-item Hamilton Rating Scale for Depression (HAMD-24); (4) have psychological pain scores ≥ 123 as measured using the Orbach & Mikulincer Psychological Pain Scale (OMMP); (5) consent to participate, and (6) can communicate and express themselves with the researchers. We changed the original inclusion criteria from “ ≥ 136” to “ ≥ 123” for OMMP scores because a later cross-sectional study on psychological pain in depressed patients conducted by our team showed that when the OMMP was ≥ 123, it suggested that patients might have suicidal tendencies.

#### Exclusion criteria

Those who have psychiatric co-morbidities, have an intellectual disability, or are undergoing other psychological therapy will be excluded.

### Sample size and recruitment

In this study, the primary outcome of the psychological pain score was selected to calculate the effect sizes. Based on the literature [20], the post-intervention psychological pain score measured using DT was 1.5 ± 0.98 for the experimental group and 2.3 ± 1.05 for the control group, with a statistically significant difference, and an effect size d of 0.788 was calculated. In the G*Power software, the number of participants in each group sample was calculated as 35 cases by using two independent samples t-test and taking bilateral α = 0.05 and 1-β = 0.9. Considering a dropout rate of 20%, the minimum sample size was therefore derived as 84 patients, 42 in each group.

Nurses will introduce the study to depressed inpatients who meet the inclusion criteria, and if a patient agrees verbally to participate, nurses will notify the researchers. The researchers will explain the information about the study in more detail to participants, discuss the appropriate timing of the intervention, and provide informed consent. Then, the participant will return the signed informed consent to the researchers. Participants will be recruited from June 2022 to June 2023, and once recruited, patients will be randomly assigned to the experimental or control group.

### Randomization and masking

Patients will be divided into two groups using simple randomization. This study will use a computerized random number generator in a 1:1 ratio to generate sequence numbers. Then, these sequence numbers will be coded and placed in sealed opaque envelopes. The researcher will divide the patients into an experimental or control group based on the sequence numbers in the envelope of their choice. The entire randomization process will be carried out by a researcher who is not involved in the intervention and data analysis. In addition, the study will also control for the possible effects of covariates by balancing baseline characteristics between the two intervention groups, which can reduce the biased results. Because this study adopts psychological interventions that make it difficult to implement blinding of participants and the researchers conducting the intervention, this study will be blinded only to the researchers assessing the outcomes.

### Intervention

#### The control intervention

Patients in the control group will be treated as usual (TAU) during hospitalization. TAU includes care for aspects of daily living, diet, safety, drug, and routine supportive psychology. Daily living care includes providing patients with a clean environment, instructing them on good personal hygiene, and exercising more. Since depressed patients are prone to binge eating or refusing to eat, patients will be instructed to eat at regular intervals and avoid stimulating foods. To ensure patient safety, nurses will strengthen the management of various hazardous materials and promptly assess the suicide risk of newly hospitalized patients and patients with changing conditions, and so on. Patients will be given health education about depression medicine and told to take their medication on time. Supportive psychological care refers to nurses listening patiently to what patients say, supporting and understanding them, encouraging them to live active life, etc.

#### The experimental intervention

Participants in the experimental group will receive TAU together with the PPSF intervention during hospitalization. The intervention is based on three dimensions of hope theory: goal cognition, pathway thinking, and agency thinking, and is supported by the use of SFBT questions during the intervention. We hope that combining positive intervention ingredients with well-used question techniques can increase positive cognition and ultimately reduce psychological pain. The PPSF intervener is a master of Nursing Specialist who has undertaken the SFBT subject training and is proficient in the practical application of the theory. Besides, the research nurse read the patient’s electronic medical records to understand the basic characteristics of the patient before intervention. The PPSF intervention will be conducted in six one-on-one, face-to-face sessions over two weeks, each lasting 30 to 40 min, along the six themes of instilling hope, agency thinking, path strategies, goal setting, cognitive reconstruction, and reinforcement of hope. Each session will focus on the theme of the positive ingredients, with the use of SFBT classical questions. Moreover, homework assignments such as breathing exercises, relaxation exercises, and positive thinking will be given at the end of each session. The detailed components of the PPSF intervention are shown in Table 1.

**Table 1:** Outline of the PPSF intervention for patients with depression

Session 1	Implementer	Format (duration)	Theme	Instillation of hope: build trust and describe psychological pain
	Research nurse	Individual, face-to-face (30-40 min)	Content	1. Encourage patients to talk about their current psychological pain and explore the stressors of psychological pain 2. Instruct patients to perform simple deep breathing exercises
			SFBT questions	1. Normal question: What is your most serious psychological pain at the moment? Many patients in our unit suffer from psychological pain. Based on your description, psychological pain is affecting you greatly and it may be more helpful for you to discuss and learn how to let go of it during this intervention time together 2. Presuppositional question: What is the goal of this session? What can I do to help you in this situation?
			Homework	1. Make a list of psychological pain on a problem list and try to get the patient to normalize the pain and pay less attention to it 2. Perform simple deep breathing exercises on their own
Session 2	Implementer	Format (duration)	Theme	Agency thinking: uncover strengths and rebuild hope
	Research nurse	Individual, face-to-face (30-40 min)	Content	1. Watch a short video about hope and actively explore positive qualities such as hope and optimism 2. Instruct patients to perform imaginary relaxation training 3. Encourage patients to talk about touching or wonderful events they experienced during their growth stage, such as childhood memories, family happiness, career achievements, etc., emphasizing past strengths and resources
			SFBT questions	1. Strength exploration: if you think of yourself as the person you just described, what do you think are her strengths? 2. Exceptional question: what stage of growth was the happiest for you and what happened to make you feel happy? 3. Positive empowerment: what do you think your growing-up experience has given you?
			Homework	1. Recall what things reflect what strengths and characteristics of their growth, list three 2. Conduct independent imagination relaxation training
Session 3	Implementer	Format (duration)	Theme	Pathway strategy: social support and active response
	Research nurse	Individual, face-to-face (30-40 min)	Content	1. Ask the patients about the way they cope with psychological pain and how they have handled similar experiences in the past 2. Discuss how family members, friends, colleagues, and others have influenced and helped the patient during the illness or when experiencing certain things
			SFBT questions	1. Relationship question: Who do you have a better relationship with? Who will be the first person to discuss it if there is a problem? 2. Response question: every life struggles to adapt to its environment and grows upward no matter what the situation is, I believe you have taken some methods, can you talk about them specifically?
			Homework	1. Seek help from nurses, doctors, patients in the ward, parents, teachers, and classmates via cell phone and record it 2. Perform 3–5 min of brief positive breathing exercises on your own
Session 4	Implementer	Format (duration)	Theme	Determination of goals: set goals and focus on solutions
	Research nurse	Individual, face-to-face (30-40 min)	Content	1. Develop feasible short-term goals for specific psychological pain based on the patient’s strengths and in consultation with the patient 2. Discuss tips to achieve the goals and refine the sessions and steps to accomplish them
			SFBT questions	1. Focused Question: what problem is being solved today? 2. Change Question: if you start to change, what is the first thing we can see about you that has changed? 3. Miracle Question: imagine that a miracle happens when you are sleeping at night and the whole house is quiet. This miracle solves the problem you have been confused about while you were asleep. When you wake up the next day, what is the sign or change that lets you know that this miracle has indeed happened and that your problem has been solved?
			Homework	Review the content of this course, schedule the short-term goals set, form a behavior schedule, and record the completion of each day
Session 5	Implementer	Format (duration)	Theme	Reconstruction of cognition: evaluate progress and think positively
	Research nurse	Individual, face-to-face (30-40 min)	Content	1. Encourage patients to share their changes and progress and, thus, inspire confidence in change 2. Reflect on the meaning of the positive impact of the disease on life
			SFBT questions	1. Complimentary question: see the change in yourself? It is really special (recommended to use after you see a noticeable change) 2. Ruling question: now you are asked to rate yourself, how many points do you rate? 3. Guide the shift in thinking: how do you think this experience has helped you?
			Homework	1. Wake up every morning and say “believe in yourself” 3 times to yourself 2. Summarize the positive effects of the disease after this intervention
Session 6	Implementer	Format (duration)	Theme	Reinforcement of hope: look to the future and enhance hope
	Research nurse	Individual, face-to-face (30-40 min)	Content	1. Objectively summarize the gains and remaining problems throughout the intervention 2. Guide patients to express their expectations for the future
			SFBT questions	What are your feelings and thoughts about these interventions? Do you have plans after this consultation? This change means a lot to you and we see your power and ability to change. In what other areas do you think there might be changes?

### Outcome and outcome measures

#### Psychological pain

Psychological pain will be measured by the Chinese version of OMMP [45]. The scale has a total of 39 items with 8 factors. This scale is a Likert 5-point scale (from “never true in your case” to “true all of the time”), with higher scores associated with more severe psychological pain. Among depressed patients, the Chinese version of OMMP had the Cronbach’s alpha coefficient and the retest reliability of 0.974 and 0.814 [45], indicating high reliability.

#### Hope

Hope will be measured by the Chinese version of the Herth Hope Index (HHI) [46]. The scale contains three factors. The HHI has 12 items that are divided into three dimensions. Each item is rated on a Likert scale of 4 (from “strongly disagree” to “strongly agree”), with items 3 and 6 being reversed scores. The higher the score, the higher the level of hope. The Chinese scale has the Cronbach’s α coefficient of 0.874 and the re-test reliability of 0.925 in depressed patients [47].

#### Cognitive distortion

The Chinese version of the Cognitive Distortions Questionnaire(CDQ) [48] is applied to assess cognitive distortions in terms of both frequency and intensity. The scale is composed of 15 items, and each item is scored on a Likert 4-point (0–3) scale, with frequency ratings ranging from “no time” to “almost all the time” and intensity ratings from “don’t believe” to “believe a lot”. The total score was 0–75; the higher the score, the more obvious the distorted cognition. The Cronbach’s α coefficient and the re-test reliability of this Chinese scale are 0.85 in patients with depression [48].

#### Depression

The Chinese version of HAMD [49] is the most commonly used scale to assess depression and can better reflect the severity of depressive symptoms. Since there are several versions of HAMD, the 24-item version widely used in this hospital will be chosen. The Cronbach’s α coefficient and the re-test reliability of this Chinese scale are 0.646 and 0.979 in depressed patients [50].

#### Suicidal ideation

Suicidal ideation will be measured using the Self-Rating Ideation of Suicide Scale(SSIOSS) [51], which contains 26 items and 4 factors. The four factors are optimism, despair, masking, and sleep, and each entry is scored as “yes” or “no”. Suicidal ideation score was obtained by adding the scores of optimism factor, despair factor and sleep factor, and the higher the score, the stronger the suicidal ideation. The total score on the scale is 26, and a score of ≥ 12 indicated that the patient had suicidal ideation. The Chinese scale has the Cronbach’s α coefficient of 0.86 and the re-test reliability of 0.87 in undergraduates [51].

It should be explained that we did not include suicidal ideation in the outcomes when we registered at Chinese Clinical Trials.gov. However, later, we decided to add suicidal ideation as a secondary outcome, considering the strong relationship between psychological pain and suicide.

#### Other information

Other information included demographic information, clinical variables of patients, and reasons for dropout. Among them, demographic information includes participants’ gender, age, religion, education level, marital status, occupation, interpersonal relationship, and family history; Clinical variables include the type of diagnosis and medication prescribed for patients with depression. These two pieces of information are collected at the baseline. The reasons for attrition will be tracked and collected by researchers during the intervention.

### Data collection

The data collection for this study will be independently accomplished by a trained nurse who will be blind to recruitment and allocation. The study will measure outcomes at 5 time points: baseline, 1 and 2 weeks (post-intervention), and 1 month and 6 months after baseline (follow-up). The schedule of the study is shown in Table 2. As depressed inpatients are commonly hospitalized for 2–3 weeks, the first 3 measurements will be handed out on the spot in the ward, filled in, and collected. The last 2 measurements will be collected by appointment with patients in the outpatient clinic. All questionnaires will be issued in paper form and will be archived by special researchers after collection. If patients leave the wards earlier than 3 weeks, they will not be allowed to complete the questionnaire 3 times in the ward, and data collection will be completed by appointing patients to the outpatient clinic. Participants in the control group will also be required to complete the measurements at the same time points.

**Table 2:** Schedule of enrolment, intervention, and outcome assessment

**Timepoint**	**Enrolment**	**Allocation**	**Post-allocation**				**Filler**
	-T_1_	T_0_ baseline	T_1_ 1st week	T_2_ 2nd week	T_3_ 1st month	T_4_ 6th month	
**Enrolment**:							
Eligibility screen	×						
Informed consent	×						
Allocation		×					
**Intervention:**							
Intervention			×	×			
control			×	×			
**Assessments:**							
Demographic information		×					
Psychological pain		×	×	×	×	×	patient
Hope		×	×	×	×	×	patient
Cognitive distortion		×	×	×	×	×	patient
Depression		×	×	×	×	×	researcher
Suicidal ideation		×	×	×	×	×	patient

### Data analysis

SPSS 25.0 will be used for statistical analysis of the data. All collected data will be processed for intention-to-treat analysis. ANOVAs and Chi-Square tests were performed to compare demographic and clinical variables, reasons for attrition, and baseline scores on primary and secondary outcomes between the groups, and significant imbalances between the two groups will be checked. Categorical variables will be explored using chi-square tests, and continuous variables will be analyzed using t-tests or Mann–Whitney U tests, thus exploring baseline differences between the two groups. The number of drop-outs and follow-up losses in both groups will be reported based on descriptive data.

This study will compare the differences in primary and secondary outcomes between the two groups at two time points: baseline and 2 weeks(post-intervention). The generalized estimating equation model (GEE) is a statistical model developed based on the generalized linear model, specifically designed to deal with repeated measures such as longitudinal data. Given the association between outcome variables at multiple time points for individuals in our study, the GEE model will be performed to compare the trends of primary and secondary outcomes at different time points (baseline, 1 week, 2 weeks, 1 month, and 6 months) in the two groups, adjusted for confounding factors of statistical differences in the baseline. The GEE model will be applied to explore the effects of time factors, intervention factors, and interactions between intervention factors and time factors on each variable between the two groups. When there is an interaction effect, then the analysis of the simple effects will be adopted. In this study, no subgroup analysis will be performed. To reduce the risk of type I errors, Bonferroni correction will be used for multiple comparisons, that is, dividing 0.05 by the number of comparisons. Thus, *P* < 0.01 represents a statistically significant difference.

### Ethical considerations

Permission for this study was granted by the Ethics Committee(2021-KY113-01) and registered at Chinese Clinical Trials.gov (ChiCTR2100048223). Ethical standards were followed throughout this study. All potential participants are informed of the benefits and risks before deciding to participate in the program, and can then decide whether to enroll. Participants are required to sign an informed consent form when they are confirmed to participate. During the intervention, participants can withdraw randomly without affecting their following treatment and nursing care. Since the study is a non-pharmacological intervention, it is unlikely to produce adverse effects, and therefore, no data monitoring committee will be established for this study. All data will be kept confidential. The findings of the study will be presented at conferences or in published journals.

### Validity and reliability

In the design of the protocol, we invited psychiatrists, psychotherapists, and nursing specialists from tertiary-A hospitals to design and validate the PPSF protocol, ensuring its scientific validity. In the implementation of the protocol, we will use a randomized controlled trial with the strictest criteria and report the progress of the study to the ethics committee every three months. Regarding data management, all collected data will be placed in a locked cabinet. Data will be accessible only to researchers.

## Discussion

Psychological pain is a distinguishable clinical symptom in patients with depression. Current research [52] on psychological pain has mainly focused on the exploration of influencing factors and the development and cross-cultural adjustment of assessment tools, leading to the neglect of psychological pain interventions. Only three studies have reported psychological pain interventions in patients with depression, and most of these interventions used CBT. However, CBT uses the improvement of negative emotions as an entry point and does not emphasize the importance of positive emotions, which often tends to reinforce the patient’s negative cognitive bias. To further fill the gap in this area and find brief and cost-effective interventions, this study designed a positive psychological intervention implemented by nurses to reduce the psychological pain of patients with depression.

As a brief-term therapy, PPSF is framed by the hope theory and guided by SFBT. Through face-to-face consultation, patients can gradually find out their own problems, pay attention to their own advantages, increase personal self-esteem and rebuild positive perception, so as to reduce psychological pain. Suppose that the PPSF intervention demonstrates effectiveness in improving psychological distress in patients with depression. In that case, it will provide better practical value to nurses to perform psychological pain interventions among patients with depression. And such the demonstration, based on a comparative, randomized study, will provide a greater level of evidence than a simple observational study. Furthermore, the creative integration of SFBT questions with hope theory in this study will help promote the development of psychological pain theory.

## Limitations

To our knowledge, this study comes with some limitations. Firstly, because the study will use a psychological intervention that will be difficult to blind patients and researchers, an assessor-blinded randomized controlled trial will be used, which may result in potential bias. Secondly, this study will involve six psychological interventions over a 2-week period, which may be too intensive, and have a 6-month follow-up, which may result in patients dropping out. Thirdly, the participants in this study were from only one psychiatric hospital, which can affect generalizability. Finally, since this study will be conducted in one hospital, this will inevitably lead to contamination between groups, affecting the results. However, if differences in findings between the two groups are finally shown, it will indicate that the PPSF intervention is still effective even in contamination.

## Conclusion

This study aims to design simple and practical psychological pain interventions from a positive psychology perspective that nurses can implement, which adds to existing research on psychological pain interventions in depression. If the PPSF intervention proves effective, it will be put in place in hospitals by nursing staff and provide a reference basis for improving psychological pain in patients with depression.

## Data Availability

Data sharing is not applicable to this article as no datasets were generated or analyzed during the current study.

## Abbreviations:

SFBT: Solution-focused brief therapy
CBB: Cognitive behavioral bibliotherapy
CBT: Cognitive behavioral therapy
PPTBCT: Psychological pain theory-based cognitive therapy
SCBT: Simplified cognitive behavioral therapy
DT: Distress Thermometer
PPSF: Psychological Pain Solution Focused
HADM-17: Hamilton Rating Scale for Depression-17
OMMP: Orbach & Mikulincer Psychological Pain Scale
TAU: Treated as usual
HHI: Herth Hope Index
CDQ: Cognitive Distortions Questionnaire
SSIOSS: Self-Rating Ideation of Suicide Scale
GEE: Generalized estimating equation
